# Quantification of microvascular change of retinal degeneration in Royal College of Surgeons rats using high-resolution spectral domain optical coherence tomography angiography

**DOI:** 10.1117/1.JBO.28.10.106001

**Published:** 2023-10-13

**Authors:** Zhen-Jie Zhang, You-Ren Wu, Yueh Chien, Yang Chen, Shih-Hwa Chiou, Shih-Jen Chen, Jia-Pu Syu, Wen-Chuan Kuo

**Affiliations:** a National Yang Ming Chiao Tung University, Institute of Biophotonics, Taipei, Taiwan; b Taipei Veterans General Hospital, Department of Medical Research, Taipei, Taiwan; c National Yang Ming Chiao Tung University, Institute of Pharmacology, Taipei, Taiwan; d Taipei Veterans General Hospital, Department of Ophthalmology, Taiwan; e National Yang Ming Chiao Tung University, School of Medicine, Taiwan

**Keywords:** optical coherence tomography, angiography, retina degeneration, Royal College of Surgeons rats

## Abstract

**Significance:**

For research on retinitis pigmentosa in humans, the Royal College of Surgeons (RCS) rat is commonly used as the primary animal model since the disease process is similar. Therefore, it is necessary to understand how the disease develops and determine whether the treatment is effective.

**Aim:**

In this study, structural and microvascular change of retinal degeneration in RCS rats was assessed non-invasively on specific dates over 3.5 months.

**Approach:**

Using a high-resolution spectral domain (SD) optical coherence tomography angiography (OCTA), the retinal degeneration in RCS rats, from day 14 until day 126, was qualitatively and quantitatively analyzed.

**Results:**

Aside from the thinning of the retina thickness starting from 2 weeks of age, blood vessels in the deep layer of the retina also began to degenerate at about 4 weeks of age. Hole structures appeared at the inner nuclear layer and the inner plexiform layer by the age of 10 weeks. Observations of abnormal angiogenesis in the choroid began by 12 weeks of age.

**Conclusions:**

We conducted a longitudinal study of retina degeneration structure and vascular changes in an RCS rat model using a supercontinuum laser based high-resolution SD-OCTA. Combined with OCTA, OCT leads to a better understanding of photoreceptor pathology as retinal degeneration by identifying tissue and vessel loss.

## 1 Introduction

Retinitis pigmentosa (RP) is a hereditary retinal degenerative disease for which there is no effective treatment, and the disease is not entirely understood. Animal eye models,^1^^,^^2^ particularly for mice and rats, are widely used in the preclinical and basic research of ophthalmology because of their short life cycles, structures similar to those of the human eye, and various available disease models. Since the Royal College of Surgeons (RCS) rat is the closest animal model to human RP, it can be utilized for preclinical study to understand how the disease develops. Furthermore, it is crucial to determine whether the treatment is effective on this basis.^3^^,^^4^

A retinal degeneration process has three main components: structural, vascular, and functional. Imaging tools can provide various information to dissect a retinal degeneration process. For example, fundus cameras provide fundus images; histology provides structural information about cut planes; electroretinography (ERG) determines cell functionality; and fluorescein angiography measures blood vessel perfusion with no depth resolution. Optical coherence tomography (OCT) has become successful in clinical ophthalmology applications due to its noninvasive, *in vivo*, and high-resolution (HR) tomographic imaging capabilities.^5^

Based upon histological examination and OCT findings in previous studies, RCS rats have been shown to exhibit progressive retinal layer thinning and photoreceptor degeneration.^6^^,^^7^ However, the limited axial resolution of conventional OCT made it challenging to interpret retinal layer structures precisely. Hence, high axial resolution is required for rodent ophthalmic imaging to distinguish all retinal layers accurately. OCT for small animals is still relatively less prevalent in preclinical research until the new development of light sources. A supercontinuum laser, a broadband superluminescent diode, and a high-speed swept laser can generate HR OCT;^7^^–^^9^ this imaging technology has gradually been favorable for assessing pathological changes in small animal eyes.

Besides, OCT angiography (OCTA) has been widely applied in clinical research because of its label-free and depth-resolved features compared to fluorescein angiography or fundus camera.^10^^–^^12^ It has been known that retinal degeneration is associated with retinal blood circulation because of the high metabolic demand of the neuroretina.^9^^,^^10^ Several clinical studies have utilized OCTA to investigate retinal diseases, such as RP, age-related macular degeneration, and diabetic retinopathy.^10^^–^^12^ Along with the advance in innovative imaging approaches, many research groups have used OCT and OCTA to investigate retinal diseases in rodent models. For example, Baumann et al. utilized multi-functional OCT and OCTA to characterize spontaneous retinal neovascularization in low-density lipoprotein-knockout mice.^13^^,^^14^ Hsu et al. used a multi-contrast polarization diversity OCT and angiography to rapidly identify disease symptoms among mouse models of different eye diseases.^15^ Tan et al. used Doppler OCT and OCTA imaging to examine the structural and blood perfusion changes in the rat retina with elevated intraocular pressure.^16^

Tan et al. reported a longitudinal structural and microvascular examination using a swept-source OCTA to decipher the pathologies of photoreceptor degeneration in RCS rats.^9^ Capillary loss in the deep capillary plexiform, quantified by vessel density, was found and was associated with the progressive dystrophy of the photoreceptors, suggesting that the progressive dystrophy of the photoreceptors is related to their microvasculature environment. In this study, we sought to use the HR spectral domain (SD) OCTA, based on the supercontinuum light source, and investigate the time course of progressive retinal degeneration in RCS rats. The retinal lesions and angiopathy were qualitatively and quantitatively monitored and analyzed weekly for consecutive weeks.

## 2 Materials and Methods

### 2.1 Animal and Experimental Protocol

The RCS rats were used in this study. The advantage of this model is that the disease process is similar to research on RP in humans. The Mertk gene defect causes retinal pigment epithelium (RPE) dysfunction, degeneration of photoreceptor cells, and vision loss. In this study, serial HR SDOCT and OCTA images were collected from 12 eyes of six RCS rats. The experiments were performed on weeks 2, 3, 4, 5, 6, 7, 8, 10, 12, and 18. For comparative analysis, serial HR SDOCT and OCTA images were collected from wild-type (WT) rats of various age, serving as a control group. H&E stain and tissue immunostaining were used for verification after the rats were sacrificed. All experiments were approved by the Institution for Animal Care and Use Committee at National Yang Ming Chiao Tung University (ethical code: #1090427).

### 2.2 Setup of HR SDOCT Imaging System

This study uses the SDOCT system developed in-house.^8^ A broadband (wavelength range: 650 to 1800 nm) supercontinuum fiber laser (SuperK Extreme, NKT Photonics, Denmark) was used as an illumination source in the present system. Using an appropriate dichroic mirror (DMSP1000−Ø1" shortpass dichroic mirror, 1000 nm cutoff) and color filter (CF) (long pass filter, FF01-715/LP-25, Semrock) set, the desired center wavelength 850 nm with full-width at half-maximum (FWHM) 240 nm was chosen for our experimental measurements. The interference signal was recorded using a spectrometer with a 2048 pixels line scan sensor with a maximum A-line scan rate of 130 kHz (CS800-800/300-250-OC2K, Wasatch Photonics). With 750  μW incident power, the system delivers an axial and lateral resolution of ∼2 and 5  μm in tissue, respectively, which allows visualizing blood vessels at an individual capillary level resolution.^17^ There are 500 A-scans in each B-scan. To achieve a higher SNR, 40  μs A-line exposure time and five repetitions at each B-scan generate one OCTA B-scan image. Therefore, 2000 cross-sectional two-dimensional (2D) images at 400 positions in one three-dimensional (3D) volume were obtained within 40 s. The enface images of the retina were obtained from B scan data using a z-projection method of ImageJ.

### 2.3 Process of OCT Structural and OCTA Imaging

**Fig. 1:**
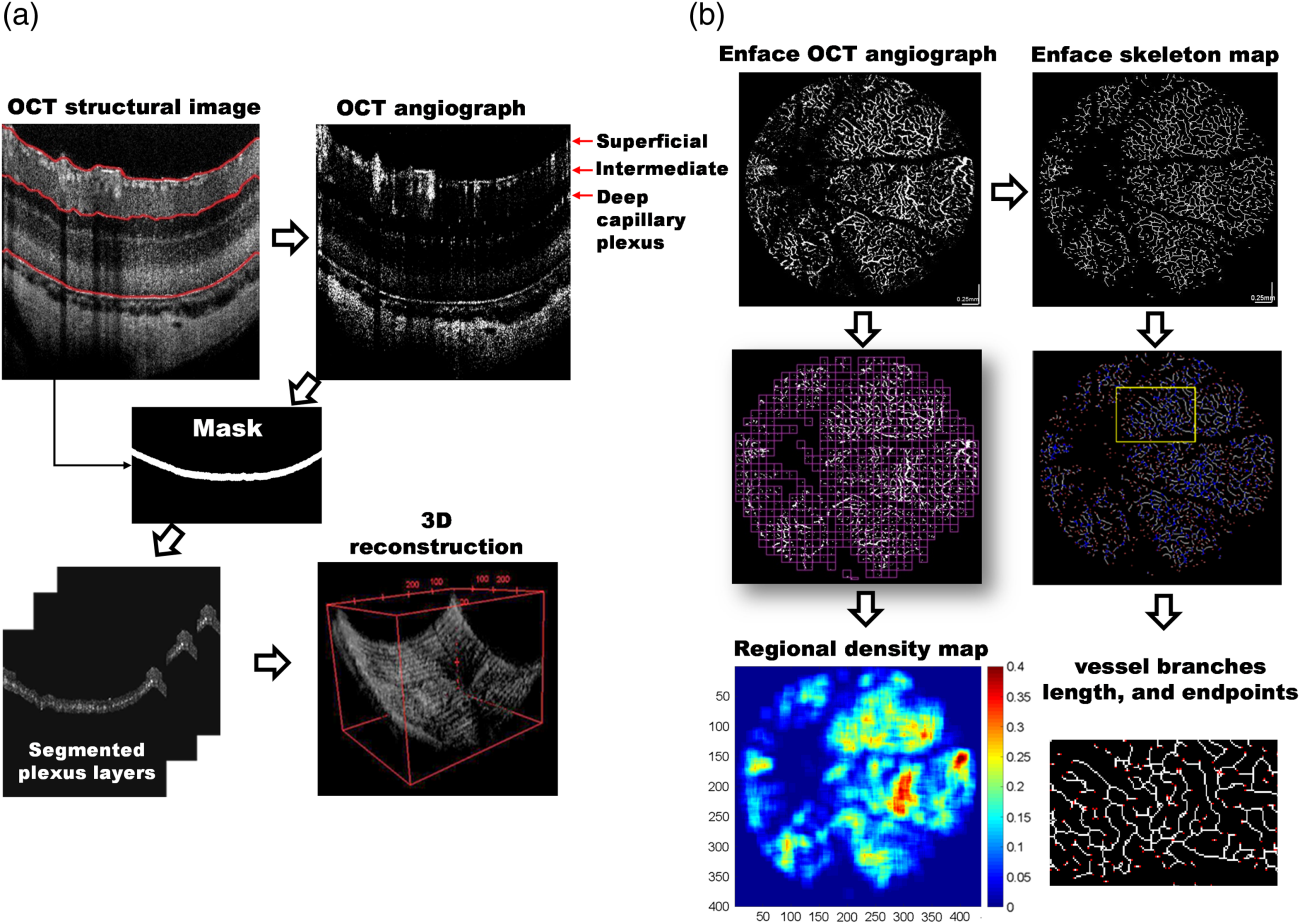
Analysis of UHR SDOCT and OCTA. (a) Flow chart of image processing and quantitative analysis: segmentation was performed to select the superficial and deep capillary plexus layer. (b) Four quantitative parameters from the enface OCTA image were analyzed, including vessel area density (ratio): area of vessels (white area)/entire area (window size 20×20  pixels), the number of vascular endpoints/area, the number of vascular branches/area, and the vessel length (in pixel unit).

As shown in Fig. 1(a), after the Fourier transform of the obtained resampling spectral interferograms, the OCT structure images were calculated and displayed on a logarithmic grayscale in a 2D format. The reflectance signal strength in each OCT image is corrected so that its intensity grayscale distribution is within a similar dynamic range before OCTA is calculated to avoid reflectance variation affecting the quality of the OCTA image. Besides, data with poor OCT image quality were excluded from this study. The OCTA images were then obtained based on calculating the difference of complex scattering signal between sequential 2D OCT images.^18^ The vessels in the rodent retina can be separated into three layers, the superficial layer within the nerve fiber layer/ganglion cell layer (GCL), the intermediate capillary networks within the inner plexiform layer (IPL), and the deep capillary networks within the outer plexiform layer (OPL).

The gradient features^19^ in each layer of the OCT retinal map are used to automatically determine three retinal layers in the OCT map (marked in red): the superficial layer, the junction between IPL and inner nuclear layer (INL), and the RPE. The superficial red line was then widened by 10 to 20 pixels to create a mask for segmenting the superficial capillary networks. Moving the center red line (i.e., IPL/INL layer) to the deep blood vessel position (i.e., OPL) and widening it by ∼10 to 20 pixels to create a mask. The location is then applied to the corresponding OCTA image to segment the deep capillary networks. It is necessary to fine-tune the shift distance manually based on different data. Finally, the superficial and deep capillary networks are generated by multiplying the masks with OCTA images. Thus, a 3D volume of OCTA was projected onto the 2D forward (enface) view by maximum intensity projection to visualize the details of the vascular change.

As shown in Fig. 1(b), vessel area density is defined as the ratio of the area of the vessels in the enface OCTA to the area of the imaging region within each window.^20^ Results were shown in color scale to highlight the regions with greater density. The same window size (20×20  pixels) was used throughout the study. The “number of vascular terminal points,” the “number of vascular branches,” and the “vessel length was counted from the vessel skeleton map.”^21^ All parameters were automatically calculated using a program written in MATLAB (MathWorks).

### 2.4 Statistics

Data are analyzed using Prism (GraphPad). One-way repeated measures analysis of variance (ANOVA) was used to detect the within-group difference in the density, slab, endpoint, and length of the retinal microvasculature over time. The Greenhouse–Geisser correction was applied to adjust the lack of sphericity in the one-way ANOVA. The tukey’s method was used as a post hoc test to correct the P value for multiple testing and to estimate the difference among the continuous measurements over the experimental periods. A P value<0.05 was regarded as statistically significant.

## 3 Results

### 3.1 Structural Change of Retina in RCS Rats

**Fig. 2:**
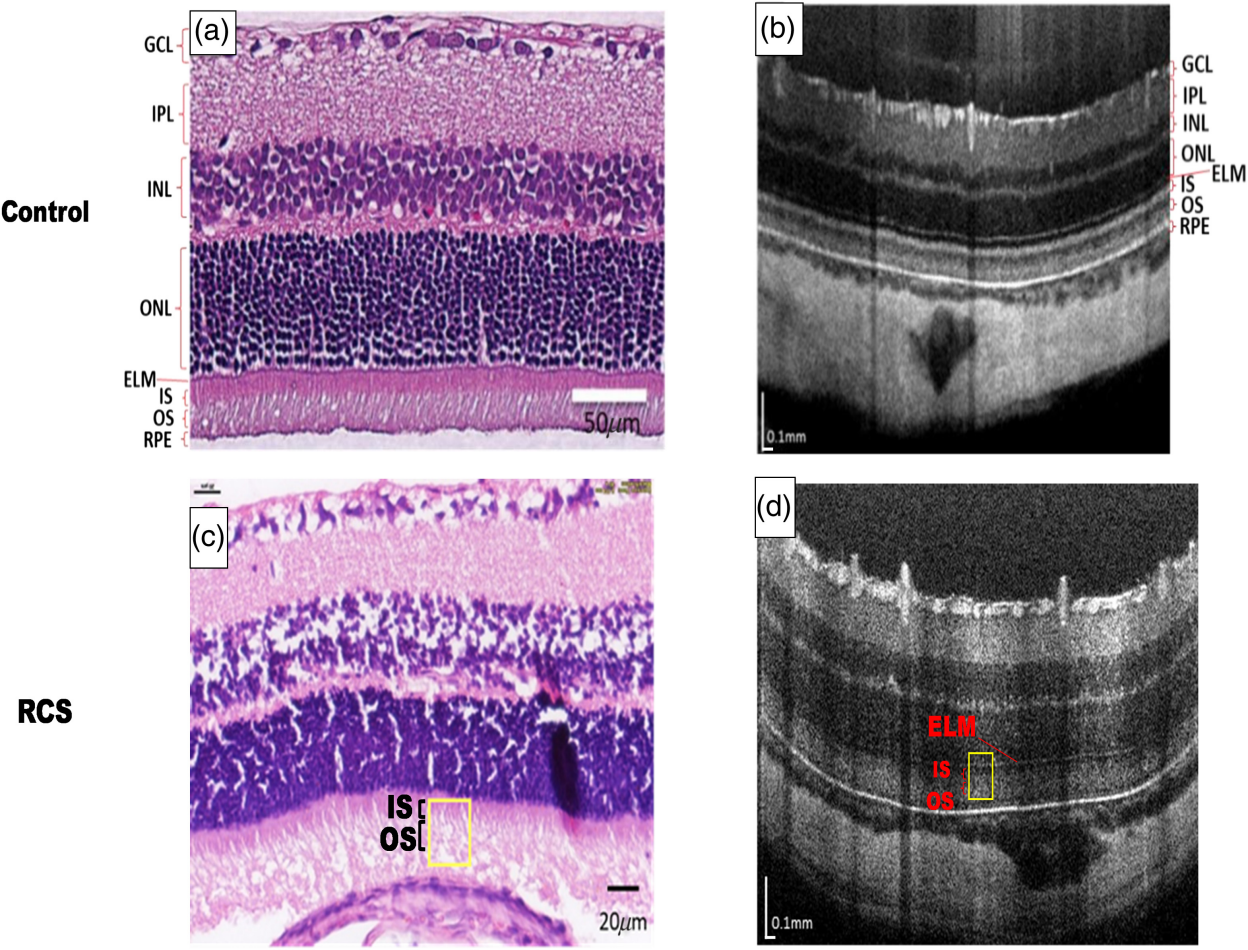
HR OCT structural images of (b) 2-week-age control (normal SD rats) and (d) RCS rats; (a), (c) their corresponding H&E stained sections. At this stage, the IS/OS was still present in the RCS rats.

Figure 2 shows an OCT image of the normal retina in SD rats at 2 weeks of age [Fig. 2(b)] and its corresponding H&E staining [Fig. 2(a)]. H&E staining revealed a normal arrangement of photoreceptor cells within the outer nuclear layer, the inner segment (IS), and the outer segment (OS) layers of OCT. HR SDOCT imaging showed that the OS layer merged with the debris layer was indistinguishable in the retinas of RCS rats, compared with that in control healthy rats. The IS layer remained unaffected, and the external limiting membrane (ELM) layer was still visible in week 2 [Fig. 2(d)]. H&E stained sections also confirmed the accumulation of debris and that the OS layer had been affected [Fig. 2(c)]. The results indicated the development of photoreceptor degeneration from an early age in RCS rats. To monitor the alterations in structural defect and angiopathy in RCS rats over time, we conducted a further longitudinal study to examine the disease progression of the affected retinas in the following experiment.

**Fig. 3:**
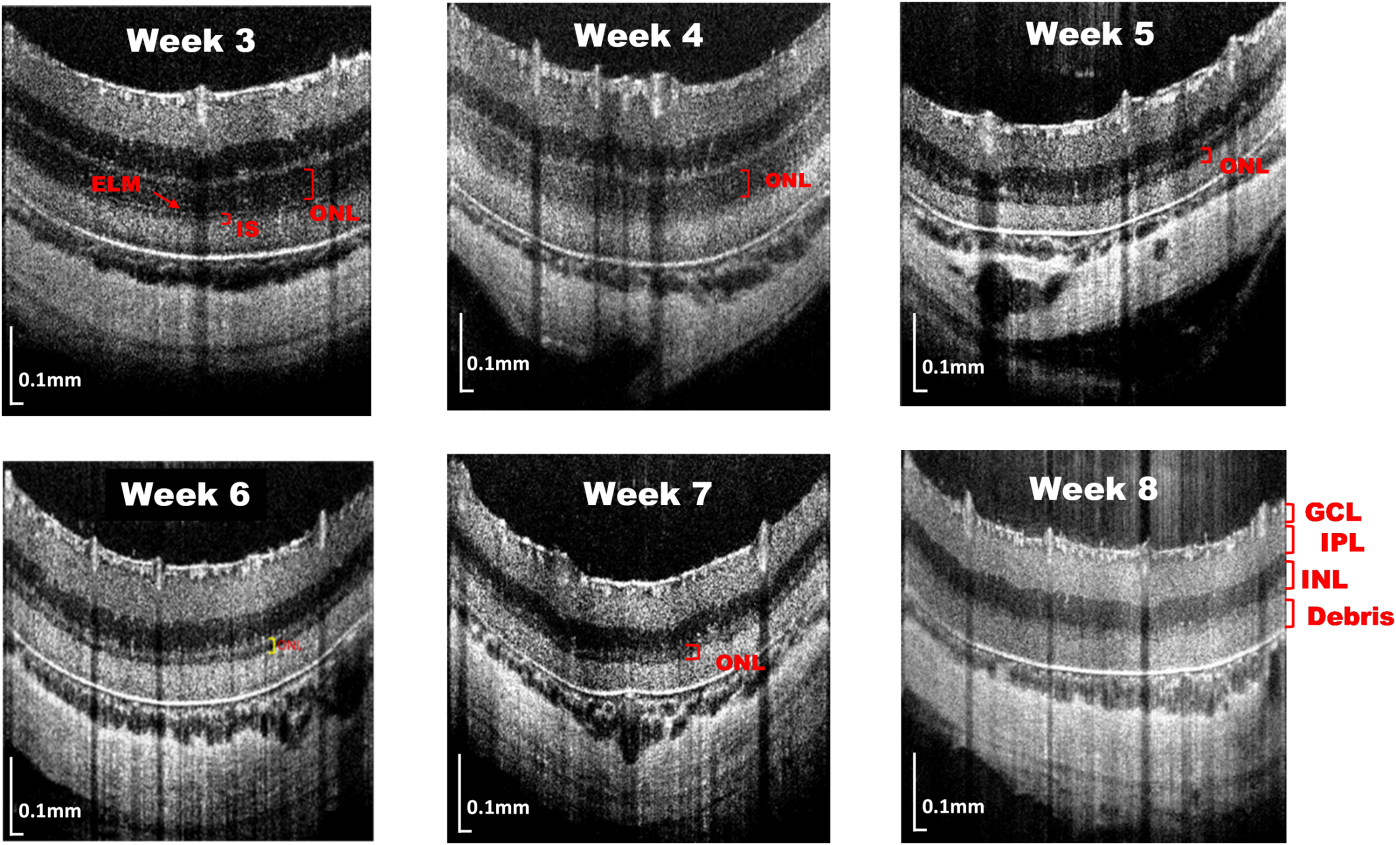
Retinal structure images of RCS rats from third week to the eighth week age. The ONL gradually became thinner and could not be separated from the debris at week 8.

In the third week, the IS thickness continued to decrease following the accumulation of debris, but ELM remained still distinguishable. However, by the fourth week, ELM was mixed with the debris layer and no longer distinguishable from it. At the same time, the low-scattering feature of the ONL changed to a high-scattering pattern. Then, the ONL gradually degenerated and disappeared until it disappeared entirely by the eighth week, resulting in the structural alterations in which the INL was adjacent to the debris layer (Fig. 3). Collectively, we used the noninvasive imaging to characterize the structural changes of the retinal layer in the RCS rats from the second week to the eighth week during the disease progression.

**Fig. 4:**
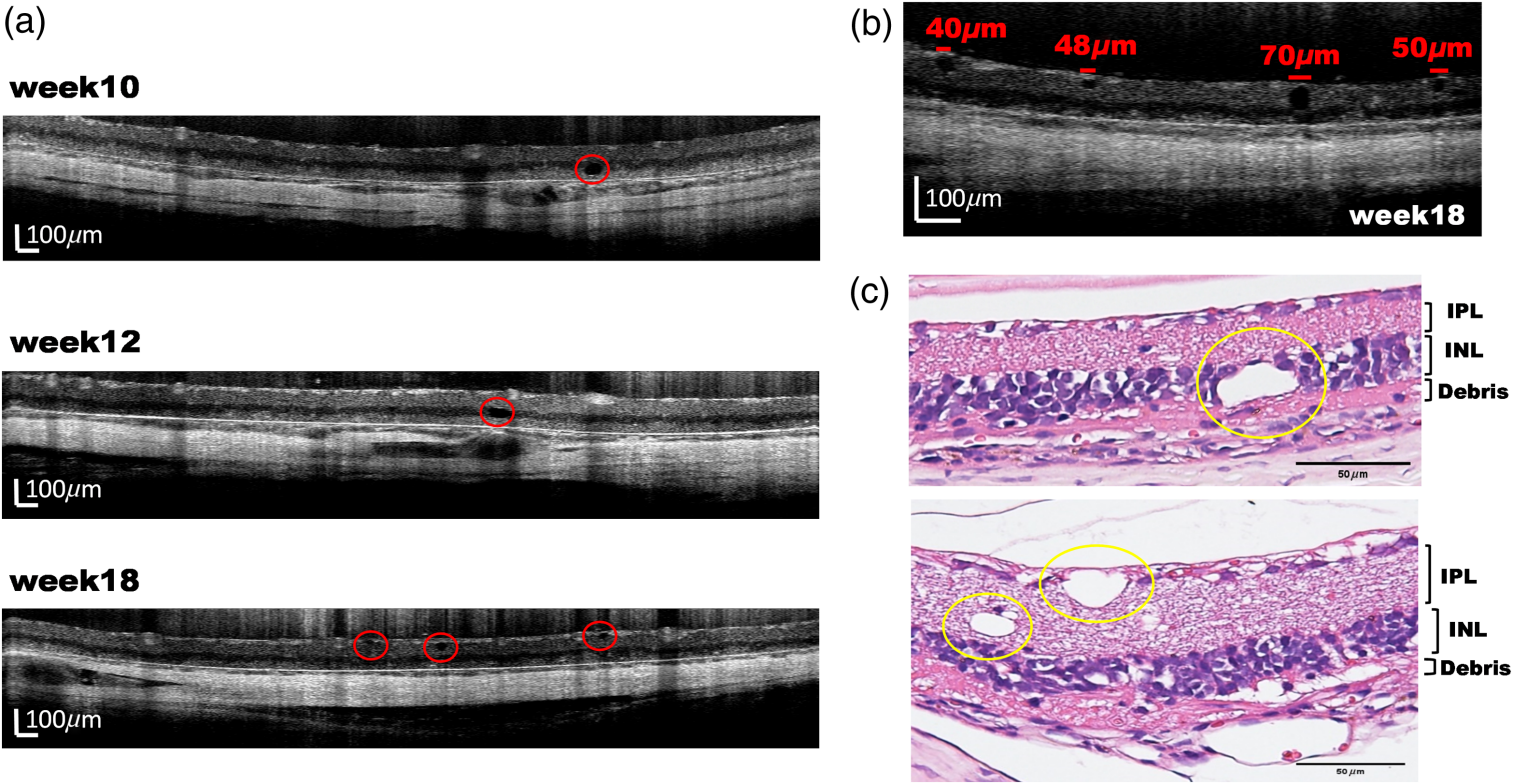
(a), (b) The structure of holes in INL, IPL, and GCL from weeks 10, 12, and 18. (c) H&E stained sections.

Figure 4(a) shows that black holes appeared in the INL and IPL in the OCT images by the 10th week. These pores appeared in the INL earlier than the IPL, and the area of the pores increased along with the weeks of age. The enlarged figure showed the pore size is about 40 to 70  μm [Fig. 4(b)] with a round and oval shape. The position, shape, and size of the pores in OCT images were consistent with the cavity structure in the H&E-stained sections [Fig. 4(c)]. Therefore, these pores were inferred as cavity structures without the cytoplasm or nucleus.

### 3.2 Microvascular Change of Retina in RCS Rats

**Fig. 5:**
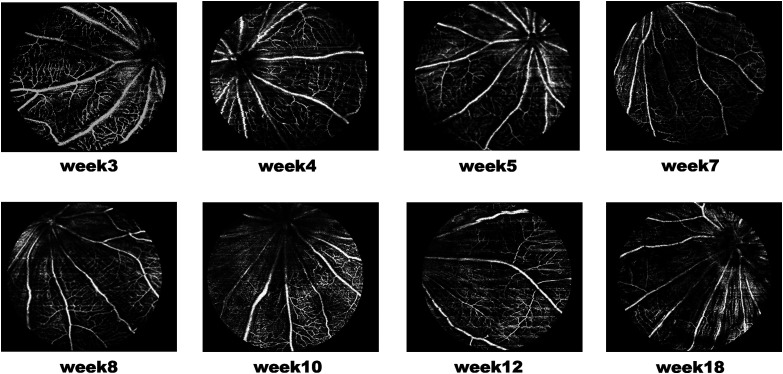
Representative enface OCTA images showing the superficial capillary plexus of RCS rat retinas from 3 to 18 weeks of age.

**Fig. 6:**
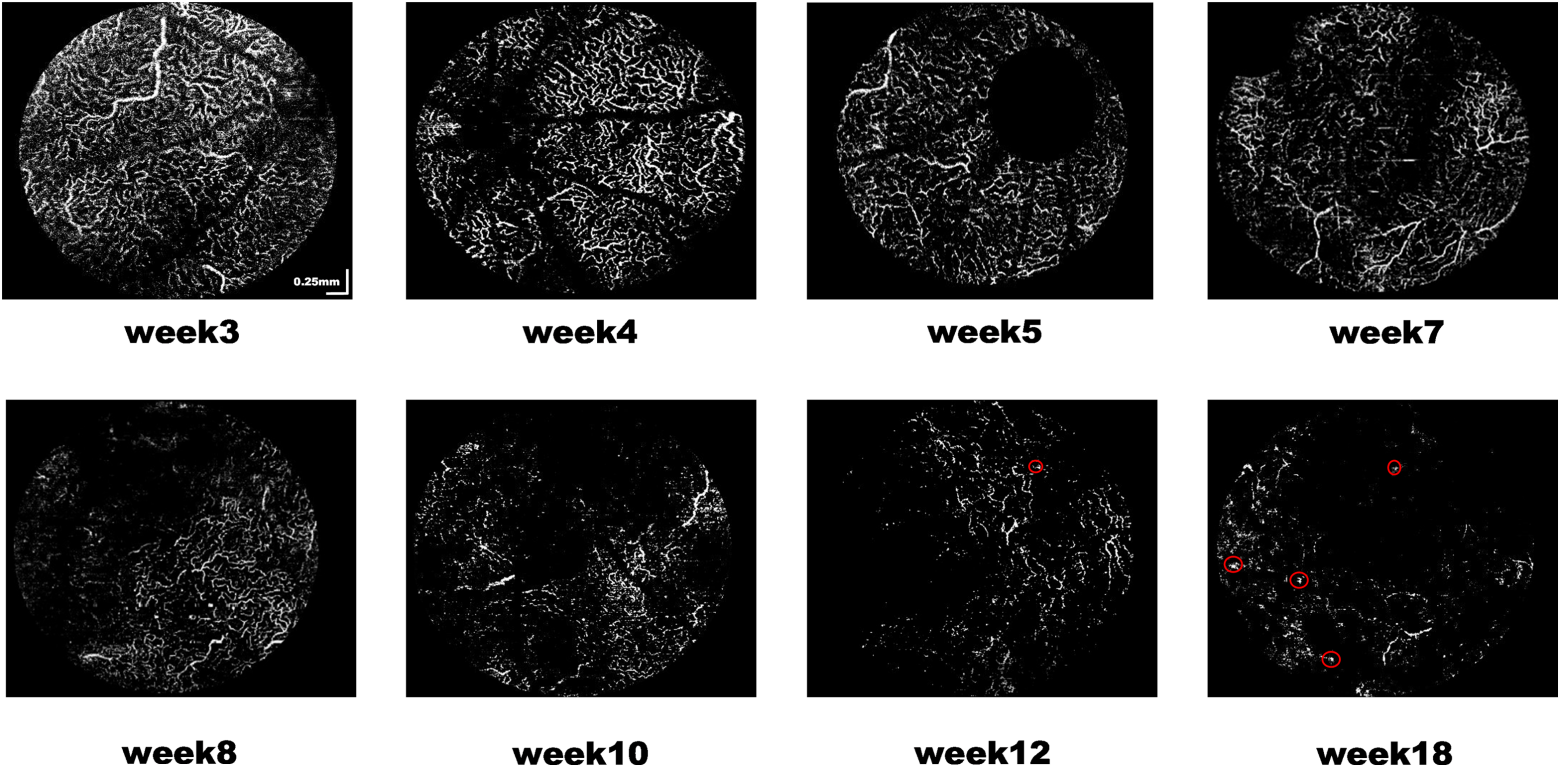
Representative enface OCTA images showing the deep capillary plexus.

In this study, superficial (Fig. 5) and deep capillary plexiform (Fig. 6) vasculature were segmented from RCS rats’ 3D OCTA data set. The OCTA projection map in the deep vascular network exhibited reduced blood vessel density and increased fragmentation. The vascular network began to shrink and degenerate, showing a disorganized pattern (red circles) by the 12 week (Fig. 6).

**Fig. 7:**
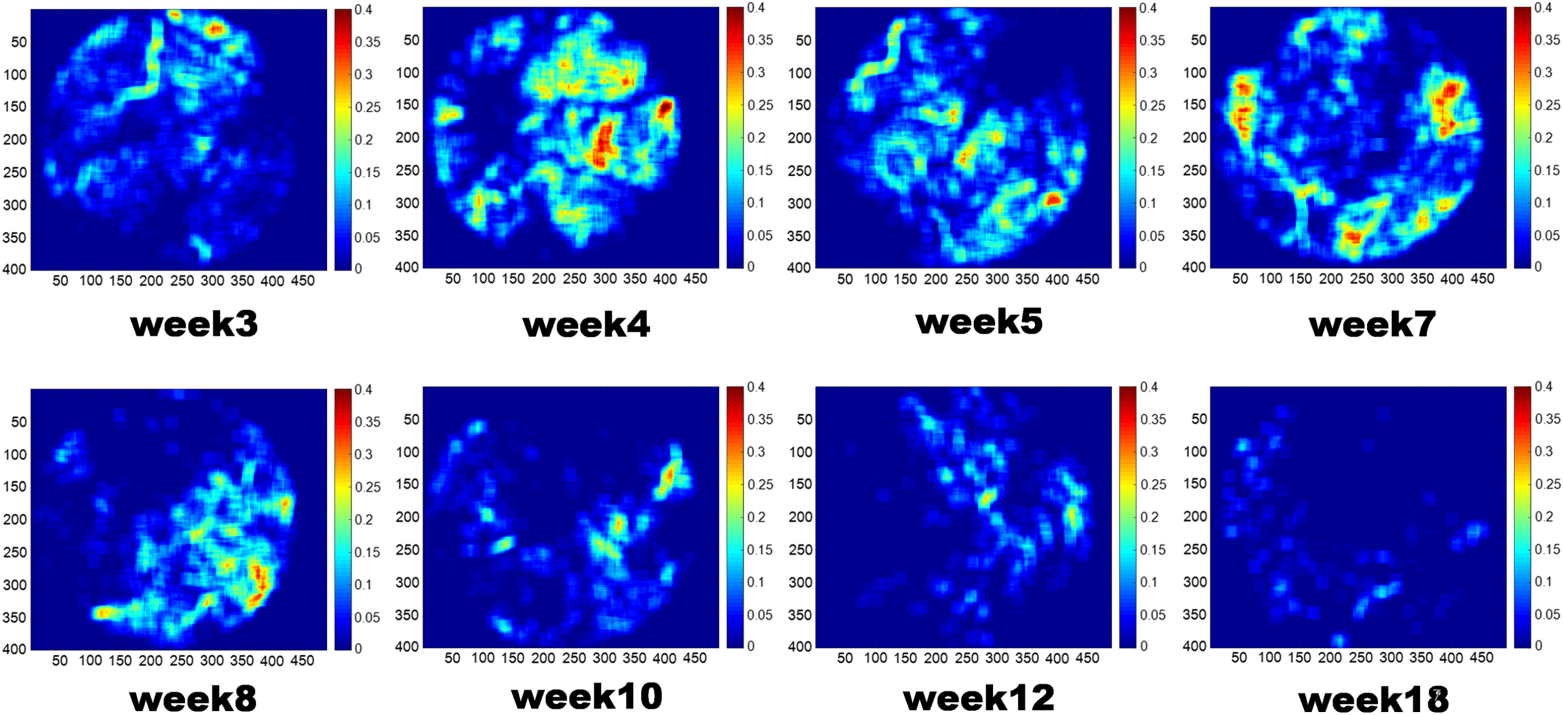
Representative area density map calculated from enface OCTA map.

**Fig. 8:**
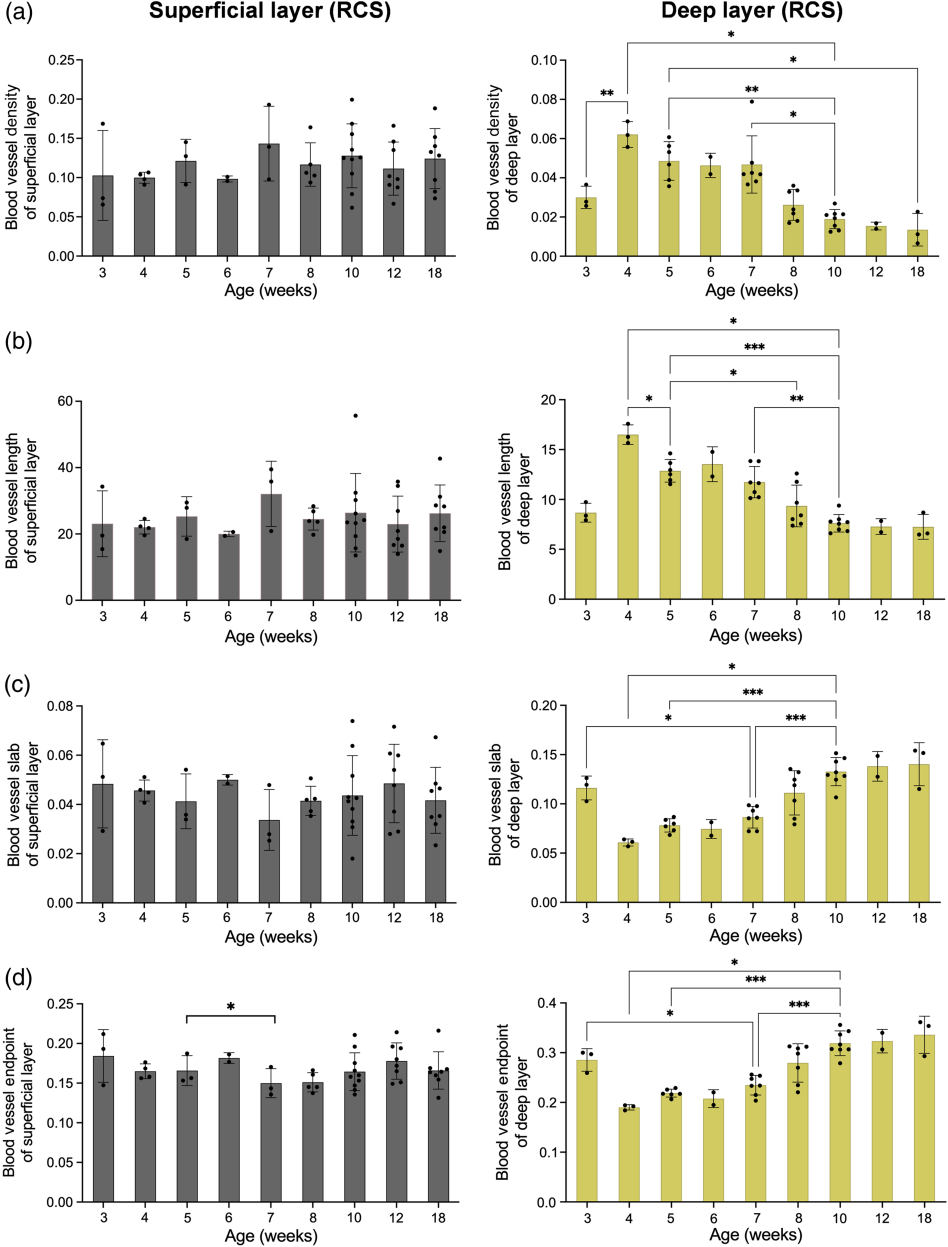
Time course of superficial (left column) and deep (right column) vasculature changes in RCS rat retinas from 3 to 18 weeks of age. (a) Vascular density, (b) the average blood vessel length, (c) the number of vessel branches/area, and (d) endpoints/area. Significant difference test: *P<0.05; **P<0.01; ***P<0.001.

**Fig. 9:**
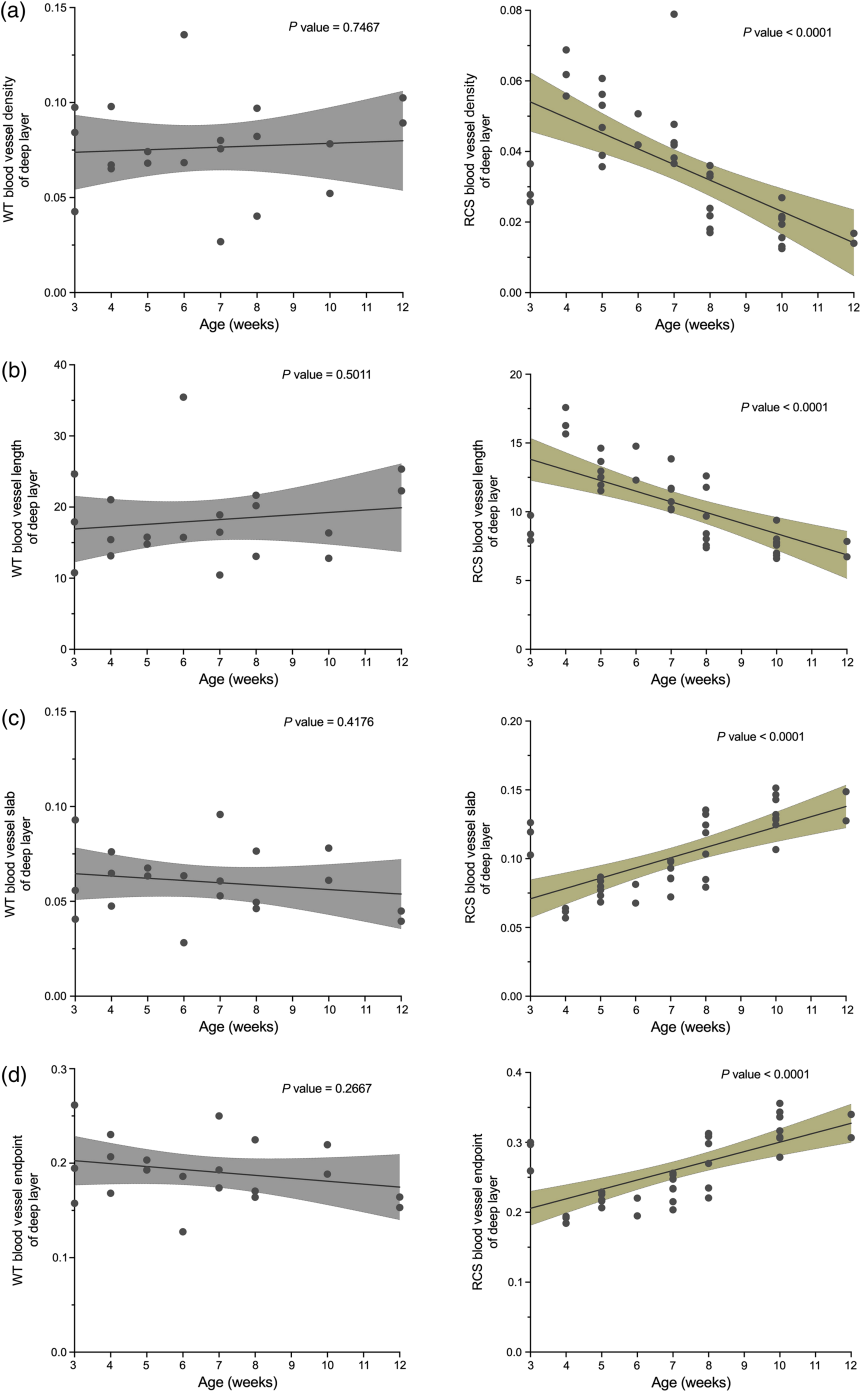
Time course of deep vasculature changes in control WT rat (left column) and RCS rat (right column) retinas. (a) Vascular density, (b) the average blood vessel length, (c) the number of vessel branches/area, and (d) endpoints/area. Linear regression was used to fit the data, and P values are shown.

To quantify the shrinkage and degeneration of blood vessels, we calculated the vascular area density map as shown in Fig. 7. Pseudo colors ranging from blue to red represented the area density from low to high. The area density was gradually declined and entirely vanished by the 18th week. Figure 8 shows the quantification graph of the time course of vasculature changes in RCS rats. According to the left column of Fig. 8, super superficial plexiform vasculature is unchanged, consistent with the previous report.^9^ Compared to the superficial vascular network, there was a significant increase in the area density from the third to the fourth week in the deep capillary plexiform [Fig. 8(a), right column]. This may due to the development of the vascular network took about a month, and the deep vascular network was not completely developed until the fourth week (Fig. 6). After the fourth week, the density gradually decreased and dropped robustly in the 10th week (p<0.05). The vessel length gradually shortened after 4 weeks, decreased significantly until the 10th week, and maintained a constant length from the 10th to the 18th week, as shown in Fig. 8(b) (right column). The quantification results of the endpoints and the numbers of vessels slab are shown in the right column of Figs. 8(c) and 8(d), respectively. The blood vessels broke down during retina degeneration due to atrophy, and the number of endpoints and slabs in blood vessels elevated as the degree of fragmentation increased. Compared to the deep vascular network in control WT rats that have no significant correlation with time in four parameters (Fig. 9, left column), there was a monotonically decreasing trend of vessel density and length over time in the deep capillary networks of RCS rat. Slab and endpoint numbers show the same increasing trend over time (Fig. 9, right column, P value<0.0001.).

**Fig. 10:**
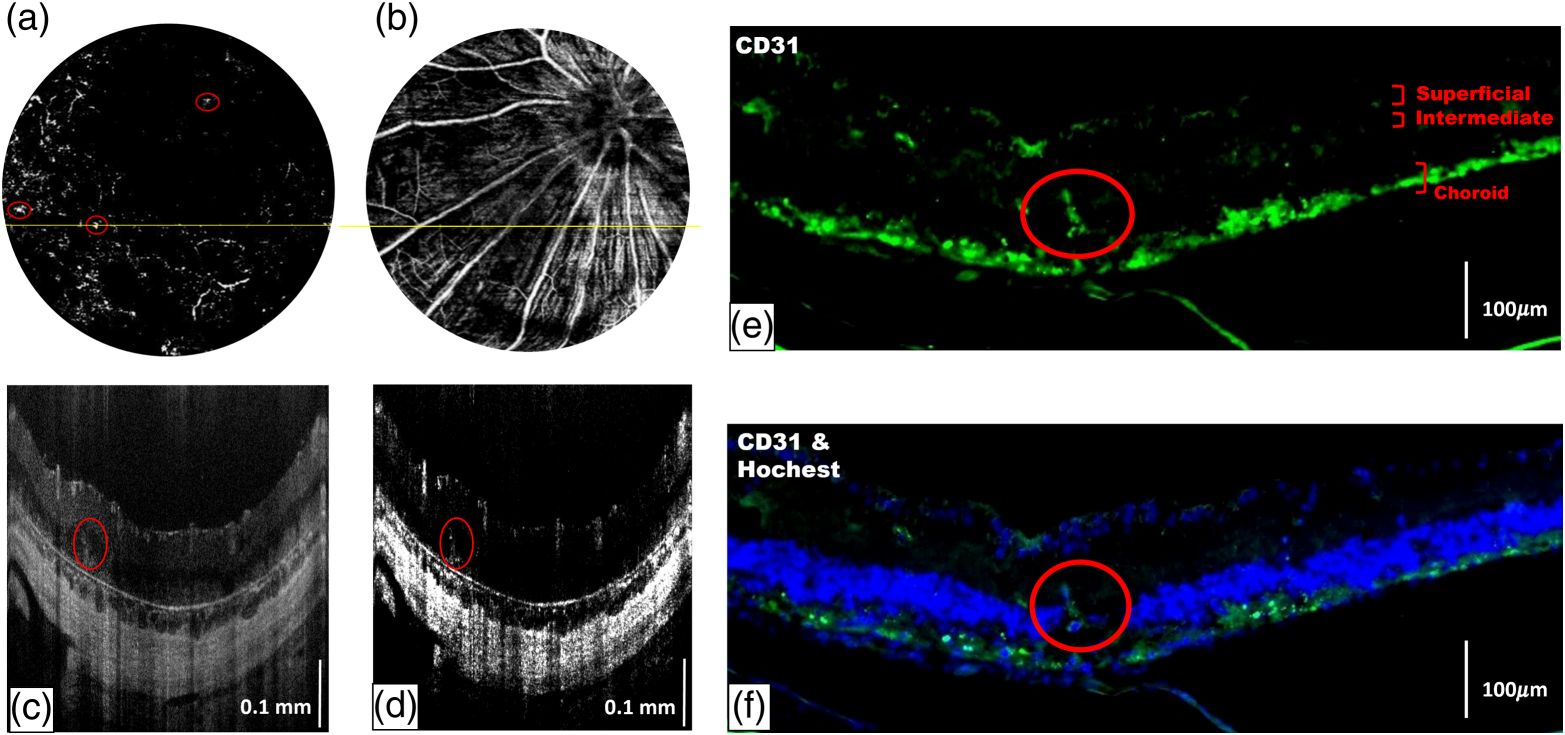
OCTA projection map in (a) deep capillary layer and (b) superficial layer of RCS rat’s retina at aged 18 weeks; (c), (d) the OCT images and OCTA cross-sections at the yellow line of (a) and (b). (e), (f) The corresponding immunostaining using CD31 (green) and Hoechst (blue). Scale bar: 100  μm. The red circle indicated that the abnormal vascular tuft grew from the choroid.

A red circle in Fig. 10(a) indicated the deep capillary plexiform with lumpy structures in the OCTA projection map of RCS rats at 18 weeks of age. As shown in Figs. 10(c) and 10(d), the bright signals indicated blood vessels as the red circles in both OCT images and OCTA cross-sections. We speculated that the abnormal neovascularization from the choroid to the retina was due to the impaired barrier functions of the RPE layer. Using large superficial vessels as landmarks [Fig. 10(b)], serial histologic sectioning was carried out horizontally and cut into several 6-μm thick sections near the position of the yellow line of Fig. 10(b) to ensure the position was identical to that of the OCT/OCTA measurement [Figs. 10(c) and 10(d)]. Immunostaining was used for verifying the results of OCTA imaging, and the anti-CD31 antibody was used to label the vascular endothelial cells. As shown in Fig. 10(e), the CD31-positive structure represented the vascular structure. The area stained positive for Hoechst included the GCL and ONL (containing the nuclei of retinal ganglion cells and photoreceptors, respectively) and the choroid [Fig. 10(f)]. Remarkably, the area that penetrated across the retinal architecture was also stained positive for both CD31 and Hoechst, indicating abnormal neovascularization in the RCS rat retinas at the late stage of disease progression.

## 4 Discussion

OCT imaging has revealed degenerative processes in the IS and OS layers of the photoreceptors of RCS rats,^6^^,^^7^^,^^9^ which correlate with ERGs and histological changes. Our study uses the HR SD-OCTA system (∼2  μm), resulting in better image quality than the previous study. We investigated the degeneration process in the RCS rat retina weekly from week 2 to week 18. The HR SD-OCT morphological images at week 2 already showed the abnormalities of the photoreceptor layer, including the debris layer accumulation and OS layer degeneration. These results agreed with those obtained from the histology sections [Fig. 2(c)]. As the age of RCS mice increased, the ONL thickness continued to decrease until that it disappeared entirely in the 8 weeks of age (Fig. 3). It is known from the previous literature^22^ that the ERG signals almost disappeared at this time, indicating that the function of photoreceptor cells is lost.^23^^,^^24^ The highly reflective materials left in the outer retinal area represented the shed debris.

Along with the increase of age, INL gradually approached the pigment epithelium and swallowed a small number of debris layers. After 10 weeks of age, an uneven structure appeared between the INL and the debris layer. The distance between the INL and the RPE decreased over time [Fig. 4(a)]. OCT images also showed empty structures in the retinal fragment layer and INL [Fig. 4(b)]. The shape, size, and location of the pores were consistent with the cavity structure seen in the H&E stained sections [Fig. 4(c)]. As the number of weeks progressed, the porous structure becomes apparent in INL and IPL, and the number tended to grow. To our knowledge, this is a novel finding of structure loss during retinal degeneration in RCS rats.

The RCS rat model is not the only popular rodent model. rd1 and rd10 mice lacking the intact pde6 β-subunit associated with mutations in the Pde6β gene can also be considered mouse models of RP. In previous SD-OCT studies with rd1 and rd10 mice, outer retinal thickness was observed to be declining steadily.^25^ SD-OCT longitudinal observations revealed that the ONL became hyper-reflective at week 3 in rd10 mice and gradually thinned thereafter.^26^ Moreover, intracellular vacuoles were also observed in the ONL of rd10 at week 3. So far, these studies focus on detecting the thickness change of the retina.

Kim et al.^27^ conducted longitudinal OCTA to characterize dynamic changes of trilaminar vascular plexuses in WT and rd10 mouse retinas. Vascular densities in all three plexuses continuously decreased with aging in both WT and rd10. However, abnormal density reduction in rd10 occurred at day 17, and the deep capillary of rd10 showed significantly low density. A recent longitudinal observation in RCS rats using swept source OCT/OCTA imaging was also proposed where Tan et al.^9^ demonstrated both structural and microvascular changes in the retina. During progressive dystrophy of the photoreceptors, the capillary loss was measured by vessel density. According to their findings, photoreceptor degeneration in RCS rats was associated with capillary loss in deeper plexuses but not superficial plexuses. In RP patients, OCT revealed similar findings,^10^ demonstrating a reduction in retinal circulation compared with normal controls. Moreover, the vascular loss was more noticeable in peripheral portions of the deeper plexuses. However, no significant differences were observed for the vessel density of perifoveal superficial layers in RP eye.

In this study, we observed the vascular network in retinal OCTA projection images from 3 weeks to 18 weeks of age in RCS rats. The deep blood vessels were found to shrink and degenerate after the fourth week when the vascular network had fully developed. Using fluorescein angiography, a previous study demonstrated the leakage of blood vessels from week 8.^28^^,^^29^ Moreover, due to the degeneration of the retinal vascular network, blood flow is reduced, stimulating angiogenesis to supply oxygen and nutrients to the tissues. As circled in Fig. 6, the projection images of blood vessels showed lumpy structures in RCS rats from 12 weeks of age. Immunohistochemistry [Figs. 10(e) and 10(f)] confirmed that abnormal angiogenesis extended from the choroid to the inner retina, which was also consistent with the observations in previous literature.^28^^,^^29^

In addition to the vessel area density, our measurements further obtained three quantitative parameters for an objective assessment of the shape in deep capillary plexiform of RCS rats, as it varied and degenerated with age: namely the “number of vascular endpoints,” the “number of vascular branches,” and the “average vessel length.” The deep vascular network was most complete in the 4 weeks of age, so there was fewer endpoints and vessels slab. A gradual increase in blood vessel fragmentation was observed from 5 weeks, with a significant difference from 10 weeks of age [Figs. 8(b)–8(d), p<0.001]. The number of endpoints and slabs in blood vessels increased as the degree of fragmentation increased. The average blood vessel length followed the area density pattern, which reached its maximum in the 4 weeks of age when the blood vessels were fully developed, then decreased due to the breakdown of blood vessels. To our knowledge, the above results are the first quantitative analysis of continual change of vascular shape per week during retinal degeneration in RCS rats.

However, there are some limitations in this study. First, the image quality and vessel distribution over the entire field may not be homogeneous, leading to spatial and temporal variation of OCT/OCTA signal. Therefore, compensating algorithms^30^ may still be needed to reduce the OCTA dependence on reflectance signal strength, the vessel density quantification of the vascular complex can be more reliable. Second, long tails on the superficial retinal vessels streak vertically down the retinal layers. This projection artifact degraded OCTA’s resolution. Due to the accumulation of projected flow in the relatively high reflectance plexiform layers, the vessel density in the deep vascular plexus may be inaccurate. However, the results of the deep vessels’ relative change and significant trends with degeneration (Fig. 9) may still be considered reliable because the superficial vessels of RCS rats did not exhibit any significant changes over time. In the future, projection suppression could be used to reduce shadow artifacts from larger superficial vessels.^10^

## 5 Conclusion

In summary, using an HR SD-OCTA platform, we conducted a longitudinal study of retina degeneration structure and vascular changes in an RCS rat model. Results were as follows. First, upon collecting debris in the second week, photoreceptor cells were affected at the same time, the OS layer started merging with the debris layer. The ELM was affected in the fourth week, and pores in the retina started appearing in the 10th week. Furthermore, the distance between the INL layer and the RPE became closer in the 10th week. These subtle structural changes can only be detected with the HR-OCT system. Second, OCTA projections indicate that vascular growth did not significantly diminish until the fourth week. This study investigated vessel fragmentation and atrophic regression quantitatively and objectively, and abnormal neovascularization was found in the 12th week. Based on the findings above, combining OCT and OCTA provides insight into the sequence of tissue loss and vessel loss, allowing for a more thorough understanding of photoreceptor pathology as retina degeneration.

## Code, Data, and Materials Availability

The data supporting this study’s findings are available from the corresponding author upon reasonable request.
